# HIV-1 Vif, APOBEC, and Intrinsic Immunity

**DOI:** 10.1186/1742-4690-5-51

**Published:** 2008-06-24

**Authors:** Ritu Goila-Gaur, Klaus Strebel

**Affiliations:** 1 Laboratory of Molecular Microbiology, National Institute of Allergy and Infectious Diseases, National Institutes of Health, 4/312, Bethesda, Maryland 20892-0460, USA

## Abstract

Members of the APOBEC family of cellular cytidine deaminases represent a recently identified group of proteins that provide immunity to infection by retroviruses and protect the cell from endogenous mobile retroelements. Yet, HIV-1 is largely immune to the intrinsic antiviral effects of APOBEC proteins because it encodes Vif (viral infectivity factor), an accessory protein that is critical for *in vivo *replication of HIV-1. In the absence of Vif, APOBEC proteins are encapsidated by budding virus particles and either cause extensive cytidine to uridine editing of negative sense single-stranded DNA during reverse transcription or restrict virus replication through deaminase-independent mechanisms. Thus, the primary function of Vif is to prevent encapsidation of APOBEC proteins into viral particles. This is in part accomplished by the ability of Vif to induce the ubiquitin-dependent degradation of some of the APOBEC proteins. However, Vif is also able to prevent encapsidation of APOBEC3G and APOBEC3F through degradation-independent mechanism(s). The goal of this review is to recapitulate current knowledge of the functional interaction of HIV-1 and its Vif protein with the APOBEC3 subfamily of proteins and to summarize our present understanding of the mechanism of APOBEC3-dependent retrovirus restriction.

## Background

HIV-1 Vif is a 23KD viral accessory protein that is required for production of infectious virus in a cell type-specific manner [1,2]. Viruses lacking a functional *vif *gene are severely restricted in their ability to replicate in non-permissive cell types when compared to wild type viruses. Non-permissive cell types include primary T cells and macrophages as well as some T cell lines (e.g. H9, CEM); other cell lines (e.g. SupT1, Jurkat, CEM-SS) exhibit a "permissive" phenotype and allow the uninhibited replication of *vif*-defective HIV-1 [3-8]. Results from heterokaryon analyses, in which permissive and nonpermissive cell lines had been fused, suggested that nonpermissive cells expressed a host factor inhibiting the replication of *vif*-defective HIV-1 [9,10]. Sheehy *et al*. subsequently identified this host factor through a subtractive cloning approach as CEM15, now generally referred to as APOBEC3G [11]. APOBEC3G is a cytidine deaminase whose natural expression is largely restricted to nonpermissive cells. Importantly, transfer of APOBEC3G into the permissive CEMss cell line or transient expression of APOBEC3G in 293T cells rendered these cells nonpermissive, thus demonstrating the critical importance of APOBEC3G in establishing a non-permissive phenotype [11].

### The APOBEC family of cytidine deaminases

**Figure 1:**
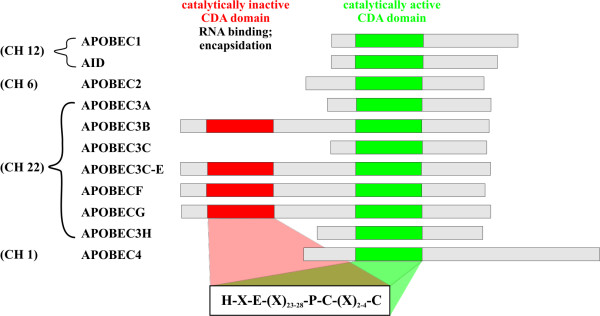
**Human APOBEC proteins**. Members of the APOBEC family contain either one or two CDA domains. Proteins are aligned based on their catalytically active deaminase domain (CDA) depicted in green. Catalytically inactive CDA domains in two-domain enzymes are depicted in red. The consensus sequence for the CDA domains is shown at the bottom. Chromosomal association is shown on the left.

APOBEC (*apo*lipoprotein *B*mRNA-*e*diting *c*atalytic polypeptide) proteins are a group of cytidine deaminases, which in humans include AID and APOBEC1 (located on chromosome 12); APOBEC2 (chromosome 6); and a series of seven APOBEC3 genes, which are tandemly arrayed on human chromosome 22 [12]. These are APOBEC3A, APOBEC3B, APOBEC3C, APOBEC3DE, APOBEC3F, APOBEC3G, and APOBEC3H (Fig. 1). Recently, a new APOBEC subfamily, APOBEC4, was identified [13]. Human APOBEC4 is located on chromosome 1 and orthologs of APOBEC4 can be found in mammals, chicken, and frogs. In mice, APOBEC4 seems to be primarily expressed in testes but its function is currently unknown [13]. In human tissues, APOBEC4 is only poorly expressed and does not appear to restrict wild type or *vif*-defective HIV-1 (Goila-Gaur, unpublished data).

APOBEC1 is an RNA editing enzyme and is the founding member of the APOBEC family of cytidine deaminases [14]; its expression in humans is restricted to the small intestine where it is involved in the regulation of cholesterol metabolism [15]. APOBEC1, in conjunction with APOBEC complementing factor, acts in a highly specific manner and normally deaminates only a single cytosine (C^6666^) on the more than 14,000 nucleotide long apolipoprotein B mRNA to create a premature translational stop codon [14,16]. However, APOBEC1 editing fidelity was found to be severely compromised when the protein was overexpressed in rat hepatomas [17]. Similarly, overexpression of APOBEC1 in transgenic rabbits and mice led to extensive non-specific editing of apoB mRNA as well as other mRNAs and was associated with liver dysplasia and hepatocellular carcinomas [18]. Finally, APOBEC1, when overexpressed in *Escherichia coli*, even deaminates DNA substrates [19] although the physiological significance of DNA deamination by APOBEC1 remains unclear. These results demonstrate that overexpression of APOBEC proteins can lead to aberrant functional phenotypes that are distinct from their normal physiological properties.

### Structural characteristics of APOBEC proteins

All APOBEC family members contain a characteristic domain structure. A short α-helical domain is followed by a catalytic domain (CD), a short linker peptide, and a pseudocatalytic domain (PCD) [12]. In APOBEC3B, APOBEC3F and APOBEC3G, the entire unit is duplicated to form the domain structure helix1-CD1-linker1-PCD1-helix2-CD2-linker2-PCD2 [12]. Each catalytic domain contains the conserved motif H-X-E-(X)_27–28_-P-C-X_2–4_-C (Fig. 1), in which the His and Cys residues coordinate Zn^2+ ^and the Glu residue is involved in the proton shuttle during the deamination reaction [12,20-22]. There is currently no high-resolution structure of APOBEC3G. This is in part due to technical difficulties with the purification of recombinant APOBEC3G, which is highly insoluble in purified form and has a tendency to precipitate during purification and concentration [23,24]. Despite these technical difficulties one recent study provided initial insights into the low resolution structure of APOBEC3G using small angle x-ray scattering [25]. The authors proposed an elongated structure for APOBEC3G that forms a tail-to-tail dimer [25]. However, this structural model of APOBEC3G is tentative and solving the high resolution structure of APOBEC3G clearly is one of the most eminent and challenging problems in the APOBEC field. An important step towards this goal was accomplished by the recent determination of the high resolution structure of the C-terminal catalytic domain (CTD; residues 198–384) of APOBEC3G [26]. It is important to keep in mind that the authors had to introduce a number of amino acid changes (L234K, F310K, C243A, C321A, and C356A) to increase protein stability and solubility of the protein although these changes did not affect deaminase activity in an E. coli-based *in vitro *assay [26]. The APOBEC3G CTD revealed a well-defined core structure of five alpha-helices (α1 – α5) and five beta-strands (β1 – β5), in which the zinc-coordinating catalytic domain encompasses helices α1 and α2 as well as the β3 strand [26].

APOBEC3G forms homo-multimers. The intrinsic propensity of APOBEC3G to multimerize was independently verified in structural and biochemical studies [12,25,27-32]. Indeed, fluorescent energy resonance transfer studies suggest that the protein is packaged into viral particles as an oligomer bound to RNA [32]. Interestingly, however, multimerization does not appear to be obligatory for APOBEC3G catalytic activity or virus encapsidation since a dimerization-deficient mutant of APOBEC3G retained both catalytic and antiviral activities [27]. Nevertheless, wild type APOBEC3G does presumably assemble into oligomeric structures under normal conditions and the question concerning the correlation between oligomeric state and biological function of APOBEC3G remains open. It is also important to point out that the ability of APOBEC3G to form homo-multimers is distinct from its ability to assemble into large multi-protein complexes of high molecular mass (HMM), which will be discussed below (APOBEC3G complexes). This is exemplified by an APOBEC3G mutant (APO C97A) incapable of homo-multimerization that nevertheless retained its ability to form large multi-protein complexes [33].

Unlike APOBEC1, which targets single-stranded RNA, APOBEC3G selectively targets single-stranded DNA. The enzyme does not deaminate double-stranded DNA or single- or double-stranded RNA nor does it modify RNA/DNA hybrids; however, APOBEC3G does bind all of these substrates more or less efficiently [12,23,34-38]. APOBEC3G preferentially deaminates cytosine residues in a CC dinucleotide context [34,35,39-43]. However, the enzyme exhibits overall significantly lower substrate specificity than APOBEC1 and deaminates the HIV-1 genome at multiple sites without apparent hot-spots. Nevertheless, there appears to be a gradient in APOBEC3G-induced hypermutation of the HIV-1 genome that increases from the 5' to the 3' end of the viral genome [34]. In fact, recent studies identified twin gradients of APOBEC3G editing with maxima mapping just 5' to a central polypurine tract (cPPT) within the integrase gene on the HIV-1 genome and 5' to the polypurine tract near the 3' LTR (3' PPT) [44,45]. In addition, the region upstream of the primer binding site near the 5'-end of the viral genome appeared to be hypersensitive to APOBEC3G editing [44]. The mechanistic basis of this phenomenon is not entirely clear; however, the observed gradients were not due to a possible polarizing effect of the PPT RNA:DNA heteroduplexes [44]. Instead, relative editing activity correlated well with the time the minus strand DNA remains single stranded [34,44]. An additional contributing factor to the observed 5' to 3' gradient could be the processive manner, in which APOBEC3G was shown to function [24].

In APOBECs carrying two deaminase domains (CD1 & CD2), generally only one domain is catalytically active while the second domain is involved in nucleic acid binding and virus encapsidation [23,27,29,46-49]. (Fig. 1). One possible exception to this rule is APOBEC3B for which one report found both deaminase domains to be catalytically active [48]; however, this remains subject to further investigation as another report did not detect catalytic activity for the N-terminal deaminase domain in APOBEC3B [50]. Interestingly, however, Hakata et al. found that in murine APOBEC3, the N-terminal rather than the C-terminal CD domain was important for catalytic activity indicating that in the murine enzyme the catalytically active and inactive domains are swapped [50].

### APOBEC3G complexes

As noted above, APOBEC3G like most members of the APOBEC family, can bind single-stranded RNA even though its substrate is not RNA but single-stranded DNA [23,34]. Indeed, the RNA binding property of APOBEC3G may be important to regulate its catalytic and antiviral activity. This is suggested by the finding that *in vitro *catalytic activity of APOBEC3G is increased in RNase-treated samples [51]. Also, APOBEC3A, which is packaged into HIV-1 virions but lacks antiviral activity, acquires antiviral activity when the N-terminal CD region of APOBEC3G is inserted into the protein [52]. Furthermore, RNA may be involved in regulating the formation of cytoplasmic APOBEC3G high-molecular mass (HMM) ribonucleoprotein complexes [33,36,51,53,54]. Such HMM complexes of APOBEC3G have been observed for endogenous APOBEC3G in T cell lines and activated primary CD4+ T lymphocytes as well as exogenously expressed APOBEC3G in transfected HeLa or 293T cells. Immunocytochemical analyses revealed a predominantly cytoplasmic localization for APOBEC3G. APOBEC3G – unlike AID or APOBEC1 – is not a nucleocytoplasmic shuttle protein. Indeed, work by Bennett et al. suggests that the cytoplasmic localization of APOBEC3G is due to the presence of a cytoplasmic retention signal located in the N-terminal region of the protein [55,56]. Interestingly, APOBEC3G was also found in punctate cytoplasmic structures identified as mRNA processing bodies (P-bodies) [36,57-59]. Furthermore, subjecting cells to stress induced the rapid redistribution of APOBEC3G into stress granules [59]. It is unclear if assembly of APOBEC3G into P bodies or stress granules is a reversible process; however, these structures are most likely part of the HMM component of cellular APOBEC3G.

Activated CD4+ T lymphocytes are highly permissive to infection by wild type HIV-1 in contrast to resting PBMC, for which a post-entry restriction to HIV-infection was observed [51]. Interestingly, there appears to be a correlation between the intracellular configuration of APOBEC3G and the cell's sensitivity to infection. In activated CD4+ T lymphocytes, APOBEC3G was predominantly found in a HMM ribonucleoprotein complex while APOBEC3G in resting CD4+ T lymphocytes was primarily in LMM configuration [51]. Analysis of APOBEC3G deaminase activity in transiently transfected 293T cells suggested that HMM APOBEC3G was less catalytically active than LMM APOBEC3G; however, deaminase activity of HMM APOBEC3G could be restored by RNase-treatment of the complexes [51]. Importantly, APOBEC3G expression is upregulated by cytokine, tumor promoter, or mitogen stimulation [60-65] and cytokine treatment of cells induced a shift of LMM APOBEC3G to its HMM conformation paralleled by increased susceptibility to HIV-infection. These observations have led to the proposal that HMM APOBEC3G is catalytically inactive and has no antiviral activity while LMM APOBEC3G is capable of executing a post-entry block to HIV infection. It is important to point out that the post-entry restriction of HIV-1 reported by Chiu et al. [51] is a Vif-independent phenomenon and is mechanistically distinct from the Vif-sensitive restriction of HIV-1 in activated PBMC. Also, the post-entry restriction in resting PBMC was not associated with DNA editing [51]. Furthermore, it has been reported that HIV-1 is able to infect resting PBMC and that infection does not require T cell activation [66,67]. Consistent with these reports, APOBEC3G-imposed post-entry restriction was not an absolute block to HIV-infection and viral DNA synthesis was evident even in unstimulated PBMC albeit with a 24 to 48 hr delay compared to activated cells [51]. Of note, while the shift of APOBEC3G from LMM to HMM conformation in activated PBMC may contribute to increased HIV-1 replication, Vif-deficient HIV-1 remains severely restricted in these cells. Unlike post-entry restriction of resting PBMC, the Vif sensitive restriction of HIV-1 in activated T cells depends on the encapsidation of APOBEC3G into viral particles in the donor cell. Taking into consideration that APOBEC3G is packaged from the LMM pool of APOBEC3G [68] these results suggest that even activated PBMC contain sufficient levels of LMM APOBEC3G to severely limit replication of Vif-deficient HIV-1. Thus, while the shift from LMM to HMM in activated PBMC abolishes post-entry restriction of HIV-1 in a Vif-independent manner, *vif*-defective virus remains unable to establish a spreading infection in activated T cells.

The ability to transition from LMM to HMM configuration is not a peculiarity of APOBEC3G but has been observed for APOBEC-1 and APOBEC3F as well [53,69,70]. Thus, the ability of APOBEC to form high molecular mass ribonucleoprotein complexes, while not necessarily relevant to the cells' ability to control HIV-1, could be important for the control of other intracellular events such as retrotransposition by retroelements. In support of this, APOBEC3G was found to inhibit retrotransposition of Alu elements through sequestering Alu RNAs in cytoplasmic APOBEC3G ribonucleoprotein complexes [71,72]. On the other hand, APOBEC3A, which does not appear to form high molecular mass multi-protein complexes, is a potent inhibitor of LTR-retrotransposons and adeno-associated virus [53,73-76] but does not generally inhibit retroviruses with the exception of Rous sarcoma virus (RSV), which is moderately sensitive to APOBEC3A [77].

### Virus encapsidation of APOBEC3G

Among all APOBEC proteins, APOBEC3G has arguably the strongest antiviral effect and most of the published work concerning the antiviral activities of APOBEC proteins involves APOBEC3G. APOBEC3G is incorporated into budding HIV-1 virions in the absence of Vif, where it mediates extensive dC to dU mutations of the minus-single-stranded viral DNA formed during reverse transcription. It has recently been shown that Vif-deficient virions produced from human PBMC contain only about 7 (+/- 4) copies of APOBEC3G [78]; yet, these virions are completely non-infectious suggesting that the level of tolerance for virus-associated APOBEC3G is quite low. In tissue culture assays packaging of APOBEC3G is roughly proportional to the intracellular expression level and transient expression of APOBEC3G in HeLa cells can lead to packaging of several hundred copies of APOBEC3G per virion (Strebel, unpublished). Not surprisingly, mutations introduced into HIV-1 genomes via deamination by transiently over-expressed APOBEC3G can be quite extensive and effectively block virus replication [34,39,41-43,79-84]. While it is obvious how the introduction of G to A mutations into a viral genome can have a negative impact on viral fitness, a number of recent studies propose additional deamination-independent activities of APOBEC proteins, in particular APOBEC3G and APOBEC3F [23,27,47,71,73-76,85-94]. However, the relative contribution of deamination-dependent and deamination-independent activities of APOBEC3G and APOBEC3F to their overall antiviral activity remains unclear. Catalytically inactive APOBEC3F showed similar antiviral potency than the wild type protein when analyzed in transiently transfected 293T cells [89]; catalytically inactive APOBEC3G, on the other hand, was generally less effective than the wild type protein [27,89]. Importantly, when wild type or deaminase-defective APOBEC3G was expressed in stable cell lines that were selected to reflect close to physiological conditions, mutant APOBEC3G exhibited no significant antiviral activity, thus highlighting the importance of enzymatic activity for APOBEC3G's antiviral effect [95,96]. It cannot be ruled out, of course, that mutation of the APOBEC3G catalytic domain induces conformational changes affecting the protein's antiviral properties. Therefore, analyzing the relative contribution of deaminase-dependent and -independent activities to the overall antiviral effect of APOBEC proteins will be a continuing effort.

**Figure 2:**
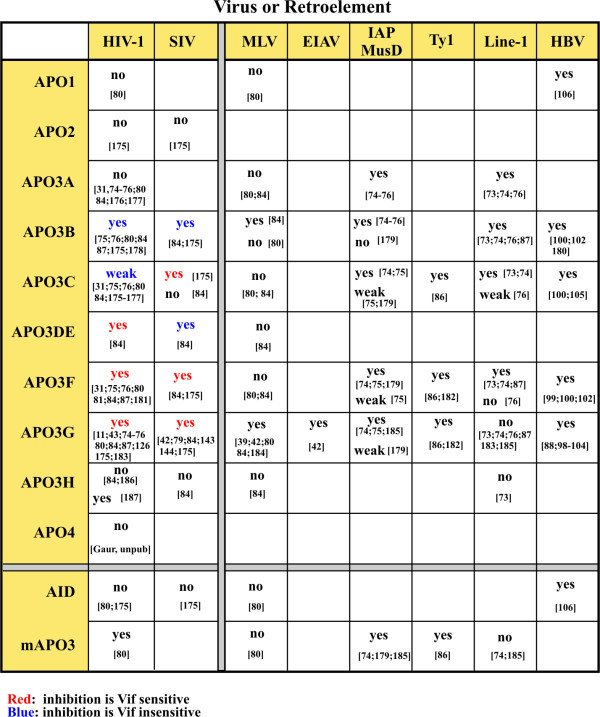
**Sensitivity of viruses or retroelements to inhibition by cytidine deaminases**. Viruses and retroelements are listed at the top and deaminases are listed on the left. Inhibition by deaminases was qualified as "no" (= insensitive to deaminase), weak (= weakly sensitive to deaminase), and "yes" (highly sensitive to deaminase). For HIV-1 and SIV viruses, the sensitivity to inhibition was further qualified as Vif-sensitive (red) and Vif-insensitive (blue). Sources of data are indicated in square brackets and include [11,31,39,42,43,73-76,79-81,84,86-88,98-106,126,143,144,175-187].

Interestingly, the antiviral effects of APOBEC3G are not limited to HIV-1 but extend to other retroviruses including murine leukemia virus (MLV), mouse mammary tumor virus (MMTV), simian immunodeficiency virus (SIV), and equine infectious anemia virus (EIAV) [39,42,79,97]. (Fig. 2). In addition, overexpression of APOBEC3G was shown to block the replication of hepatitis B virus, a hepadnavirus whose life cycle includes the reverse transcription of an RNA pregenome [88,98-106]. Packaging of APOBEC3G into such diverse viruses suggests that virus encapsidation is either a relatively nonspecific process or involves signals shared by these viruses. In that respect it is of interest that even though APOBEC3G targets single stranded DNA it nevertheless binds RNA [12,23,34,37,38,107-109] and was found to interact with the viral Gag precursor protein through its NC component [42,79,107,110-113]. *In vitro *studies using purified recombinant NC and APOBEC3G found that the two proteins do not competitively bind RNA but instead form an RNA-protein complex [23]. The nucleic acid binding properties of APOBEC3G are associated with its two deaminase domains. While the C-terminal deaminase domain provides catalytic activity and thus engages single-stranded DNA, the N-terminal deaminase domain is catalytically inactive but may be important for RNA binding and encapsidation into virions [23,27,29,50,114]. The interaction of the N-terminal deaminase domain with RNA may also be a critical requirement for the encapsidation of APOBEC3G into viral particles although this is still an ongoing debate. Several studies suggested that viral RNA or RNA in general is not a prerequisite for APOBEC3G packaging; however, most of these reports studied virus-like particles rather than whole virus [110-112,115-117]. It is conceivable that the parameters governing encapsidation of APOBEC3G into virus-like particles differ from those for encapsidation into virions. Arguments for the involvement of viral RNA come from the observation that helper viruses and virus-like particles lacking genomic RNA package about one third of the APOBEC3G found in normal *vif*-deficient virions [107,117]. Of note, when packageable viral RNA was provided *in trans*, APOBEC3G packaging was restored to wild type efficiency [107]. Importantly, APOBEC3G packaged into helper virus in the absence of viral RNA was not associated with the viral core; however, addition of viral RNA *in trans *restored core association of APOBEC3G [37,107]. These observations suggest that viral RNA enhances encapsidation of APOBEC3G and promotes core-association. A separate line of research has investigated the role of cellular RNA and implicated 7SL RNA in the RNA-mediated encapsidation of APOBEC3G [38]. 7SL RNA is normally a component of signal recognition particles (SRP); however, it is also an abundant component of HIV-1 virions [37,38,118]. Interestingly, while the majority of 7SL RNA present in a cell is associated with SRP components, only the 7SL RNA but not the SRP components were identified in virion preparations. Indeed, overexpression of SRP19 reduced the packaging of 7SL RNA in a dose-dependent manner but could be counteracted by overexpression of exogenous 7SL RNA [38]. The absence of SRP components from HIV-1 virions suggests a specific packaging mechanism for 7SL RNA. Yet, the parameters determining the packaging of 7SL RNA are still debated. One group has identified the NC component of the viral Gag precursor as the packaging determinant for 7SL RNA [38,108]. while others did not observe a requirement for NC in the packaging of 7SL RNA [37,118]. In the latter case, minimal Gag constructs lacking NC were found to package normal levels of 7SL RNA [118]. Also, helper virus carrying a deletion of a putative packaging signal or virus lacking functional NC zinc finger domains did not package viral genomic RNA; such particles only incorporated background levels of APOBEC3G but packaged normal levels of 7SL RNA [37]. These data suggest that 7SL RNA may be necessary but is not sufficient for the efficient packaging of APOBEC3G.

### Vif-induced proteasomal degradation of APOBEC3G

**Figure 3:**
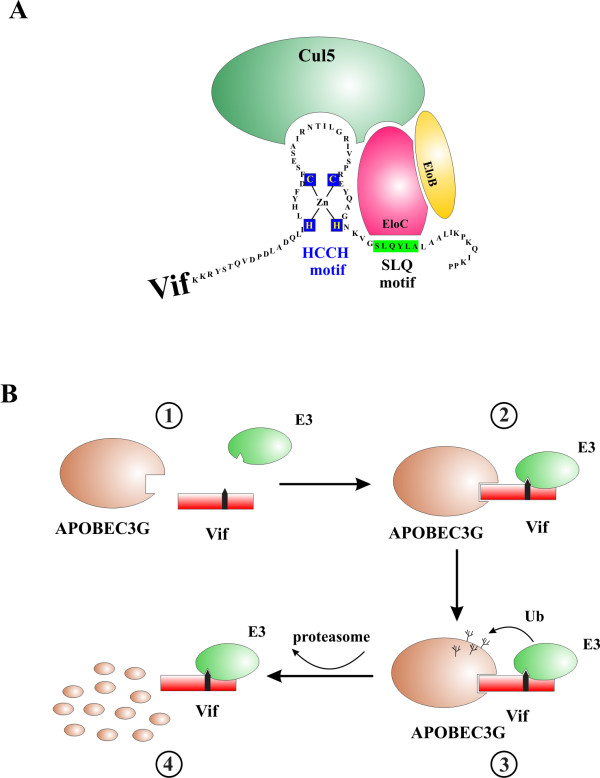
**Model for Vif-induced degradation of APOBEC3G**. **(A) **Sequence motifs in Vif implicated in the assembly of a Cul5-E3 ubiquitin ligase complex. Two conserved domains in Vif, the HCCH motif and the SLQ motif are involved in binding Cul5 and elongin C (EloC). Vif coordinates one zinc molecule, which may be required to stabilize a structure important for the binding of cullin 5 (Cul5). **(B) **Adaptor model for Vif-induced APOBEC3G degradation. According to this model Vif is an adaptor molecule with binding sites for APOBEC3G and the Cul5-E3 ligase complex (1). Expression of Vif results in the formation of an APOBEC3G-Vif-E3 ternary complex (2). This triggers poly-ubiquitination of APOBEC3G (3) resulting in the degradation of APOBEC3G (4).

The antiviral activity of APOBEC3G is strongly inhibited by Vif allowing the virus to replicate virtually unimpaired in APOBEC3G-positive host cells. Other APOBECs are targeted by Vif as well although there are significant differences in the relative sensitivity to Vif (Fig. 2). Also there is a significant species-specificity that allows Vifs from some viruses to target APOBECs from certain host species but not others (Fig. 2). Some of the Vif:APOBEC relationships remain controversial; however, there is general agreement that the inhibition of APOBEC3G's antiviral activity by Vif is mediated through a physical interaction with APOBEC3G that results in the exclusion of the deaminase from virions. This effect of Vif is generally accompanied by a reduction of the intracellular steady state levels. Over time, expression of Vif can result in a striking depletion of APOBEC3G in HIV-1-infected T cells while APOBEC3G mRNA levels remain unaffected. APOBEC3G is an inherently stable protein. In transiently transfected HeLa or 293T cells its half-life was calculated to > 8 hr and pulse/chase analyses revealed that Vif reduced the half-life of APOBEC3G to between 5 minutes and 4 hr depending on the experimental setup [119-122]. This change in APOBEC3G stability has been attributed to degradation by the cellular ubiquitin-dependent proteasome machinery [31,81,119-126]. The mechanism of APOBEC3G degradation by Vif has been extensively studied and is now relatively well understood (Fig. 3). Accordingly, degradation of APOBEC3G is triggered by a physical interaction with Vif. Several domains in Vif critical for this effect have now been identified (Fig. 3A). One of the domains involved is a highly conserved motif near the C-terminus of Vif, referred to as the SLQ motif. The SLQ(Y/F)LA sequence resembles a conserved motif in the BC box of the suppressors of cytokine signaling (SOCS) proteins and was found to mediate binding of Vif to elongin C [119,124,127,128]. a homolog to the yeast Skp1 protein and a known component of E3 ubiquitin ligase complexes. In addition, a highly conserved H-X_5_-C-X_17–18_C-X_3–5_-H motif (also referred to as HCCH motif) located upstream of the BC box was found to mediate interaction with cullin-5 [127-129]. Furthermore, two cysteine residues that are part of the HCCH motif are critical components of a zinc finger domain [130-133]. Zinc binding appears to be important for Vif function since chelation of zinc inhibited HIV Vif activity presumably by affecting the proper folding of the protein [132,134]. The HCCH domain together with the SLQ motif enable Vif to recruit an ubiquitin ligase (E3) complex containing elongin C, elongin B, cullin-5, and Rbx1 [124,127,128,130]. It is believed that binding of Vif-Cullin-5/elonginB/elonginC/Rbx1 complexes to APOBEC3G accelerates polyubiquitylation of the deaminase and, as a result, targets APOBEC3G for destruction by the 26S proteasome [120-124,127,128]. (Fig. 3B). The ability of Vif to induce polyubiquitylation of APOBEC3G was supported by *in vitro *studies in which Vif coexpressed with cullin-5, elongin B, elongin C, and Rbx1 assembled into a functional E3 ubiquitin ligase complex and induced polyubiquitination of immunopurified APOBEC3G *in vitro *[135]. These findings are contrasted by a recent study demonstrating that APOBEC3G lacking all lysine residues was nevertheless sensitive to degradation by Vif [136]. The authors propose that polyubiquitination of Vif may in that case provide the signal necessary for targeting APOBEC3G to proteasomal degradation. However, the precise mechanism of degradation of lysine-free APOBEC3G by Vif remains to be investigated.

Vif itself is a relatively unstable protein with a half-life of ~30 minutes and – like APOBEC3G – is degraded by cellular proteasomes [137,138]. It is interesting that the turnover rates calculated for Vif and APOBEC3G do not match [128](Strebel unpublished). Also, neither deletion of the SLQ motif nor mutation of the HCCH motif in Vif, both of which abolish APOBEC3G degradation, increased the stability of Vif or prevented its polyubiquitination (Strebel, unpublished) suggesting that Vif is not degraded through the cullin-5 E3 ubiquitin ligase complex. Thus, while there is solid evidence that Vif can induce polyubiquitination and degradation of APOBEC3G by recruiting the Cul5-E3 ubiquitin ligase complex, it seems unlikely that Vif and APOBEC3G are co-degraded in this complex and the mechanism of Vif degradation remains an open question.

### Degradation-independent Inhibition of APOBEC3G

Intracellular degradation of APOBEC3G clearly contributes to the exclusion of APOBEC3G from viruses. However, when a virus first infects a cell it faces high levels of APOBEC3G and the amounts of Vif produced by the virus are – at least initially – very low. Given that Vif affects APOBEC3G steady-state levels in a dose-dependent manner it can be assumed that the rate of APOBEC3G depletion in newly infected cells is directly proportional to the amounts of Vif expressed in these cells. Taking into account the fact that kinetic studies determining the half-lives of APOBEC3G were all done at relative excess of Vif [119-122]., it is unlikely that progeny virus produced early following infection is made in an APOBEC3G-free environment. Thus, if the only function of Vif were to induce degradation of APOBEC3G, virus produced early on would likely be less infectious than virus produced later on when intracellular levels of APOBEC3G have been depleted by degradation. Such a phenomenon has, however, not been observed. In fact, there is increasing evidence that Vif has additional functional properties that prevent the encapsidation of APOBEC3G into virions in a degradation-independent manner. The most striking observation in that respect is the recent identification of a degradation resistant form of APOBEC3G [33]. Degradation resistant APOBEC3G was still packaged into *vif*-deficient HIV-1 virions and had antiviral properties. Surprisingly however, Vif prevented the packaging of this APOBEC3G variant and restored viral infectivity [33]. Furthermore, the efficiency of Vif-induced APOBEC3G degradation does not necessarily correlate with the efficiency of preventing APOBEC3G encapsidation suggesting that these two effects can be functionally separated [126]. In fact, in some cases Vif was able to prevent encapsidation of APOBEC3G without apparent intracellular degradation while at the other extreme, a fluorescently tagged Vif protein efficiently caused degradation of APOBEC3G but failed to restore viral infectivity [126]. YFP-Vif alone did not affect viral infectivity excluding the possibility that the lack of infectivity in the latter example was caused by non-specific toxicity of the tagged Vif [126]. Other, more subtle observations also point to degradation-independent functions of Vif. For instance, while encapsidation of APOBEC3G into *vif*-deficient virions is generally proportional to the intracellular expression level, reduction of virus-associated APOBEC3G by Vif was in some cases significantly more pronounced than the concomitant reduction of intracellular APOBEC3G levels [79,126,139,140]. Moreover, mutation of a serine residue at position 144 in Vif (S144A) did not affect its ability to induce APOBEC3G degradation yet severely impaired Vif's ability to govern the production of infectious viruses from APOBEC3G-expressing cells [128]. Finally, Vif was found to inhibit enzymatic activity of APOBEC3G as well as the B-cell specific Activation-Induced Deaminase (AID) in a bacterial assay system [141,142]. Interestingly, inhibition of APOBEC3G and AID by Vif in the bacterial system was sensitive to mutation of residue D128 in APOBEC3G or the corresponding D118 in AID, which in other experiments was shown to affect physical interaction of Vif and APOBEC3G [143-146]. Since E. coli lacks a proteasomal degradation machinery, these results suggest that Vif can affect the enzymatic activity of APOBEC3G in the absence of proteasomal degradation. It is unclear, how Vif inhibits encapsidation of degradation resistant APOBEC3G or how it inhibits the *in vitro *deaminase activity of the enzyme in the E. coli assay. Inhibition of deaminase activity by steric interference cannot be ruled out; however, a non-functional Vif variant carrying a mutation in the HCCH box required for assembly of the Cul5 E3 ligase complex was still capable of interacting with APOBEC3G *in vitro *yet did not inhibit deaminase activity [141,142]. Two other possible mechanisms can therefore be envisioned: (i) Vif prevents packaging of APOBEC3G through competitive binding to a common packaging signal; support for this model comes from the observation that the parameters for encapsidation of APOBEC3G and Vif into HIV-1 virions are very similar (Strebel and Khan, unpublished); (ii) Vif promotes or accelerates the transition of APOBEC3G from LMM to HMM conformation. Support for the second model comes from the recent observation that Vif can induce conformational changes in APOBEC3G and promote the assembly of APOBEC3G into HMM complexes *in vitro *and *in vivo *[54]. It is also possible that both mechanisms co-exist; however, mechanistic details of the degradation-independent exclusion of APOBEC3G from HIV-1 virions have yet to be worked out. In summary, current data suggest that Vif has functional properties that can prevent the packaging of APOBEC3G and inhibit its catalytic activity through degradation-dependent as well as degradation-independent mechanisms.

### Vif/APOBEC interactions

The ability of Vif to block the antiviral activity of APOBEC3G is species-specific [79,147,148]. The Vif proteins of HIV-1 and SIV_Agm _can inhibit APOBEC3G of their natural hosts but are not known to target APOBEC3G of other species. Accordingly, HIV-1 Vif is unable to neutralize the antiviral activity of African green monkey (Agm) or rhesus APOBEC3G. Conversely, SIV_Agm _Vif is unable to neutralize human or macaque APOBEC3G. Thus, the Vif proteins of HIV-1 and SIV_Agm _function in a highly species-specific manner. The Vif protein of SIV_Mac _on the other hand acts more broadly and is able to neutralize APOBEC3G proteins from humans, African green monkeys, and rhesus macaques [79]. Several independent studies found that a single amino acid residue at position 128 in human APOBEC3G was responsible for the species specificity and change of this residue from the human to the Agm sequence (D128K) was sufficient to reverse sensitivity to HIV-1 and SIV_Agm _Vif [143-146]. Most studies found that mutation at position 128 severely affected the binding of APOBEC3G to Vif [143-145]. As far as Vif is concerned it was observed that amino acid changes near the N-terminus (residues 14–17) affected the species-specific interaction with APOBEC3G [149,150]. In addition, mutation of residues 40 to 44 in HIV-1 Vif were found to affect interaction with APOBEC3G [150,151]. and deletion of residues 43–59 abolished APOBEC3G interaction [57]. In fact, deletions in multiple regions of Vif can lead to a loss of interaction with APOBEC3G [121]. Interestingly, our own studies show that deletion of residues 23–43 in Vif had no effect on APOBEC3G interaction. The fact that in some cases amino acid changes in Vif appeared to have a more pronounced effect on APOBEC3G interactions than deletion of the same region suggest conformational constraints. Thus, residues 40–44 may be critical for proper folding of Vif but are unlikely to constitute the entire APOBEC3G binding site. Consistent with this, interference studies using overlapping Vif peptides demonstrate that residues 33 to 88 in Vif are important to form a non-linear binding site for APOBEC3G [151]. These results are supported by our own data showing severe loss of interaction with APOBEC3G for Vif mutants carrying deletions of residues 38 to 69 and 77 to 125, respectively (Strebel, unpublished). Finally, Vif was reported to form oligomeric structures. Dimerization was shown to involve residues 156 to 164 near the C-terminus of Vif and was found to be important for its biological activity [152,153]. Indeed, a peptide antagonist to Vif dimerization increased encapsidation of APOBEC3G into Vif+ HIV-1 virions suggesting that Vif oligomerization is important for interaction with APOBEC3G [153].

### Mechanism of APOBEC-mediated inhibition of viral infectivity

While catalytic activity of APOBEC3G undoubtedly is important for its antiviral effect, the precise mechanism that leads to inhibition of viral infectivity remains unclear. Hypermutation of viral genomes clearly is detrimental to HIV-1 spreading infections as mutations in the viral structural and non-structural proteins can lead to replication defects at multiple levels. However, there is still an ongoing discussion on whether editing of the viral genome can explain all the phenomena associated with infection by APOBEC-containing *vif*-defective HIV.

One possible alternative/additional mechanism to the accumulation of debilitating mutations in the viral genome is the degradation of uracilated viral cDNA through the activity of cellular DNA glycosylases, e.g. UNG and SMUG1. Degradation of nascent viral cDNAs would explain the efficient inhibition of HIV-1 in single round infectivity assays, which often only require *de novo *synthesis of HIV-1 Tat, an inefficient target for APOBEC3G due to its size, base composition, and location in the viral genome. Mutations in structural genes (i.e. gag, pol, or env) would not affect the readout of a single cycle assay because such mutations would only gain weight during progeny virus production in multi-round replication assays. Thus, the impact of editing on APOBEC3G-imposed restriction of HIV-1 *in vivo *may be underestimated by single-cycle infectivity assays. Nevertheless, degradation of nascent viral cDNAs would explain early observations on the role of Vif for the production of full-length viral reverse transcripts [7,154-156]. However, the functional interplay of APOBEC and DNA glycosylases is far from clear. One previous report found that the nuclear form of UNG (UNG2) is packaged into HIV-1 virions through an interaction with Vpr to modulate the viral mutation rate independent of APOBEC3G [157]. Another study concluded that Vpr, in fact, reduces the packaging of UNG and SMUG into HIV-1 virions by inducing their proteasomal degradation [158]. A third study did not observe any effect of Vpr on UNG packaging [159]. Consistent with this study, a fourth study reported that UNG packaging was, indeed, Vpr independent and instead involved an interaction with the HIV-1 integrase [160]. Thus, the mode of UNG packaging remains under discussion; however, most studies agree on the presence of UNG2 in HIV-1 virions. Nevertheless, the role of UNG in APOBEC3G mediated restriction of HIV-1 remains unclear. Kaiser et al found that the presence or absence of active UNG in donor or target cells had no impact on the antiviral activity of APOBEC3G [159]. Also, UNG2 appeared to be absent from highly purified HIV-2 or SIVmac239 virions [161] suggesting that, if at all, UNG2 would function in a virus-specific manner. Furthermore, overexpression of the UNG inhibitor Ugi in virus producing cells did not impair APOBEC3G function [82,159]. Finally, experiments in chicken fibroblasts lacking SMUG1 activity did not reveal an effect of UNG or SMUG1 on APOBEC3G mediated restriction of HIV-1 or Rous Sarcoma virus [162]. These observation are contrasted by studies reporting that (i) Vpr-mediated incorporation of UNG2 into HIV-1 particles is required to modulate the virus mutation rate and for replication in macrophages [163] and (ii) that virion-associated UNG-2 and apurinic/apyrimidinic endonuclease are involved in the degradation of APOBEC3G-edited nascent HIV-1 DNA [164].

When considering these seemingly contradictory reports, one must keep in mind that most of these studies involved transiently transfected APOBEC3G. Transient expression of APOBEC3G can lead to deaminase-dependent and deaminase-independent antiviral activity [89,95]. This raises the possibility that some of the seemingly conflicting results on the role of UNG or SMUG are due to deaminase-independent effects of APOBEC3G. Deaminase-independent effects of APOBEC3G on HIV-1 could potentially mask deaminase-dependent effects involving UNG1 or SMUG1, especially if APOBEC3G is expressed at high levels (see chapter on deaminase independent activities of APOBEC3G below). Also, mammalian cells contain at least two additional glycosylases, TDG and MBD4, capable of removing uracil from DNA (reviewed in [165]). UNG and SMUG1 are capable of targeting uracil in single- and double-stranded DNA while TDG and MBD4 glycosylases selectively target double-stranded DNA [166]. Given those substrate specificities it is unlikely that TDG or MBD4 are involved in APOBEC3G-dependent degradation of uridylated viral DNA. However, it cannot be ruled out that mammalian cells contain other yet unidentified DNA repair mechanisms with single-stranded DNA specificity.

### Deaminase independent activities of APOBEC3G

Previous reports of APOBEC3G-induced hypermutation have correlated cytidine deaminase activity with antiviral function [30,42,43]. There have been multiple recent reports indicating that the antiviral activity of APOBEC3G can be dissociated from its cytidine deaminase activity [47,85,89]. The deamination-independent inhibition of viral replication appears to be multifaceted and there is no clear consensus yet on this topic. For instance, one group reported that APOBEC3G and APOBEC3F inhibit the annealing of tRNA(3)(Lys) to viral RNA thereby interfering with tRNA-primed initiation of reverse transcription [91-93] while another group did not observe an effect of APOBEC3G on tRNA primer annealing but instead found that the protein significantly inhibited all RT-catalyzed DNA elongation reactions [90]. APOBEC3G also was implicated in the inhibition of strand transfer reactions [82,94]. and APOBEC3G and APOBEC3F were found to inhibit DNA synthesis and integration [83]. All of these effects of APOBEC3G and APOBEC3F were deamination independent and were observed for the most part with transiently transfected APOBEC3G or purified recombinant protein. Again, the field is not unified as one report found that catalytically inactive APOBEC3G inhibited viral DNA synthesis and integration significantly less efficiently than wild type APOBEC3G [82]. Similar to these donor cell specific effects, the reported post-entry restriction of HIV-1 in resting PBMC also did not appear to involve hypermutation of *de novo *synthesized viral DNA and thus may have been deaminase independent [51]. Finally, some of the antiviral effects of APOBEC proteins towards HTLV-1 and Hepatitis B virus did not require deaminase activity [99,104,167,168]. Nevertheless, other reports did identify APOBEC-induced hypermutations in Hepatitis B genomes using sensitive detection methods [98,100,105,169]. Again, most of these studies involved cells expressing experimentally increased levels of APOBEC3G and the importance of APOBEC proteins for the control of HTLV and Hepatitis B *in vivo *at physiological APOBEC levels remains to be evaluated.

### APOBEC family of proteins as therapeutics

APOBEC proteins and in particular APOBEC3G can provide potent intrinsic immunity to infection by viruses whose life cycle involves a single-stranded DNA step. Unfortunately, some viruses including HIV have evolved countermeasures to bypass the APOBEC3G mediated immunity. In the case of HIV-1, the viral accessory protein Vif inhibits the encapsidation of APOBEC3G and thus enables the virus to escape its antiviral effect. Inhibition of APOBEC3G encapsidation by Vif, whether it happens through proteasomal degradation or via a degradation-independent mechanism, requires the physical interaction of Vif and APOBEC3G. Thus, disrupting the Vif/APOBEC3G interaction by small molecule inhibitors or through pharmacological approaches has tremendous potential for antiviral therapy and is an active area of research. Nevertheless, there has been concern that targeting APOBEC3G could have the opposite effect and, in fact, favor the virus under conditions where APOBEC3G activity is only partially inhibited. It was argued that while a highly effective Vif inhibitor may result in mutational meltdown of the viral quasispecies, a partially effective Vif inhibitor may accelerate the evolution of drug resistance and immune escape due to the codon structure and recombinogenic nature of HIV-1 [170]. This view is supported by a recent report studying the effects APOBEC3G on viral isolates containing partially defective Vif alleles. The authors identified spontaneous drug resistance in viruses that had recombined with presumably defective hypermutated viral genomes [171]. Thus, suboptimal Vif function or partial inhibition of the Vif/APOBEC3G interaction through pharmacological methods can indeed result in the evolution of an HIV-1 quasispecies capable of rapidly adapting to environmental challenges. Nevertheless, it is important to keep in mind that the drug resistance variants that arose in the study by Mulder et al came from superinfection by wild type virus and resistant viruses invariably contained wild type Vif alleles. This could suggest that HIV-1 cannot survive long-term with partially active Vif or with partially inhibited Vif/APOBEC3G interactions. Thus, there is reason to be optimistic about the potential efficacy and safety of drugs targeting APOBEC3G.

Another approach to bypass the effects of Vif is to eliminate the Vif-sensitive N-terminal domain of APOBEC3G and target APOBEC into HIV-1 cores as a fusion to Vpr. This approach was successfully applied by Aguiar et al. who fused APOBEC3A to Vpr and found that the chimera was efficiently packaged into cores of HIV-1 and SIV virions and potently inhibited viral replication in a Vif-independent manner [172]. Aside from inhibiting the Vif/APOBEC interaction or bypassing Vif function by removing Vif-sensitive domains, decreasing the Vif:APOBEC ratio by boosting cellular expression of APOBEC3G could offer another way to outmaneuver HIV-1. APOBEC3G expression can be upregulated several fold by interferon treatment in human PBMC, primary human brain microvascular endothelial cells, and primary macrophages [60,64,173]. In addition, APOBEC3A expression is highly responsive to interferon treatment of human monocytes and monocyte-derived macrophages and APOBEC3A expression was inversely correlated with susceptibility to HIV infection [174]. Similarly, interferon-alpha treatment of human hepatocytes resulted in increased expression of APOBEC3G [101,102]. However, a subsequent study concluded that the induction of antiviral cytidine deaminases did not explain the inhibition of hepatitis B virus replication by interferons [106]. Thus, much work remains to be done before we fully understand the intricate details of APOBEC regulation and antiviral activities.

## Concluding Remarks

Since the identification of APOBEC3G as the primary target of Vif activity, research on cytidine deaminases has exploded and APOBEC proteins are arguably among the best studied cellular enzymes. We now know that APOBEC proteins are central to an intrinsic host defense mechanism that has evolved over millions of years. Thus, Vif/APOBEC3G interactions provide an important new target for antiviral therapy. Much progress has been made; however, there is a long way to go before we can claim to fully understand the complex interactions of HIV with APOBEC proteins. Obtaining a high-resolution structure of APOBEC3G and Vif for instance will be crucial to intelligent drug design. There are promising signs that the technical problems that have hampered progress until now in the structural area can be overcome in the near future.

## Authors' contributions

RG and KS equally contributed to this work.
